# Prognostic Impact of Untreated Chronic Coronary Artery Obstruction After Surgery for Aortic Regurgitation

**DOI:** 10.3390/jcdd13030115

**Published:** 2026-03-03

**Authors:** Xin Li, Vito Domenico Bruno, Yi Jiang, Yunxing Xue, Dongjin Wang

**Affiliations:** 1 Department of Cardiovascular Surgery, Nanjing Drum Tower Hospital, Chinese Academy of Medical Sciences & Peking Union Medical College, Graduate School of Peking Union Medical College, Nanjing 210008, China; lixin_doctor2000@163.com (X.L.); drjiangy@pumc.edu.cn (Y.J.); 2 Institute of Cardiothoracic Vascular Disease, Nanjing University, Nanjing 210008, China; 3 Department of Minimally Invasive Cardiac Surgery, IRCCS Galeazzi Sant’Ambrogio Hospital, 20157 Milan, Italy; vitodomenico.bruno@grupposandonato.it; 4 Department of Cardiovascular Surgery, Nanjing Drum Tower Hospital, The Affiliated Hospital of Nanjing University Medical School, Nanjing 210008, China

**Keywords:** valve disease, aortic regurgitation, coronary artery disease

## Abstract

Background: The optimal management strategy for 50–70% chronic coronary artery stenosis in patients undergoing aortic valve surgery for aortic regurgitation (AR) remains controversial. This study evaluates the prognostic impact of chronic coronary obstruction severity on surgical outcomes and mid-term survival. Methods: This retrospective cohort study included 717 patients undergoing aortic valve surgery for AR, grouped by coronary stenosis into <50% (*n* = 641) and 50–70% (*n* = 76). Following 1:1 propensity score matching (72 patients per group), the primary outcome of major adverse cardiovascular events (MACE) and the secondary outcome of all-cause death were compared. Results: No intergroup differences emerged in perioperative mortality (1.32% vs. 1.56%, *p* = 1.000) or complication rate. With a median follow-up of 2.53 years, 50–70% coronary obstruction does not increase MACE (HR = 2.050; 95% CI 0.375–11.197; log-rank *p* = 0.397) and all-cause mortality (HR = 0.710; 95% CI 0.200–2.522; log-rank *p* = 0.595). Similar results were obtained in the competing risk regression and multivariable analyses. Conclusions: In patients with AR, 50–70% chronic coronary obstruction does not increase perioperative complications, MACE, and all-cause mortality.

## 1. Introduction

Approximately 50% of patients undergoing aortic valve surgery are present with coronary stenosis, but there is still controversy regarding the management of coronary arteries in this population [1]. The ESC/EACTS guidelines for valvular heart disease recommend that coronary artery bypass grafting (CABG) be conducted in patients with coronary artery stenosis of ≥70%. For 50–70%, the recommendation and evidence level remain suboptimal [2]. Studies have shown that in patients undergoing aortic valve replacement (AVR) for aortic stenosis (AS) with coronary artery disease (CAD), concomitant CABG surgery for patients with moderate (50% to 70%) CAD reduces the risk of late death by more than one-third and does not increase operative mortality [3]. However, the Thoracic Surgery Association database also shows that the surgical mortality rate for valve surgery combined with CABG is significantly higher than that for isolated valve surgery [4]. Patients with valvular heart disease and coronary artery stenosis of 50–70% who do not undergo coronary artery surgery do not experience an increase in major adverse cardiovascular events (MACE) or mortality over a 6-year period [5]. Notably, the aforementioned studies either focused solely on patients with AS or were limited to small sample sizes and lacking control group [6,7,8].

Current guidelines are oversimplified and are not subdivided for valvular lesions. Aortic regurgitation (AR) and AS have different pathophysiological processes. In the setting of AR, prolonged volume overload leads to left ventricular dilatation and eccentric hypertrophy, thereby limiting diastolic myocardial perfusion and increasing left ventricular mass and oxygen demand. Decreased diastolic pressure in aortic regurgitation further exacerbates inadequate diastolic myocardial perfusion [9,10,11]. Therefore, AR may also be dependent on the coronary blood supply. However, considering competitive blood flow and improved coronary blood supply following valve surgery, the need for a more aggressive revascularization strategy remains controversial [12,13].

The present analysis assessed the impact of varying degrees of chronic coronary obstruction on perioperative complications, MACE, and all-cause mortality in patients undergoing surgery for AR.

## 2. Materials and Methods

### 2.1. Study Design

We retrospectively screened patients over the age of 18 years who underwent aortic valve surgery for at least moderate AR at the Nanjing Drum Tower Hospital from January 2018 through December 2024. Exclusion criteria were a history of myocardial infarction(MI), prior percutaneous coronary intervention (PCI) or CABG, prior aortic valve surgery, active endocarditis, moderate or greater AS, no preoperative coronary angiography, and concomitant a parallel coronary revascularization procedure in this surgery [14]. A total of 717 patients met enrollment criteria and were divided into two groups according to the extent of CAD (*n* = 76, 50–70% group vs. *n* = 641, < 50% group). This study was approved by the Ethics Committee of Nanjing Drum Tower Hospital (reference number: 2025-0179-01). The need for informed consent was waived as this was a retrospective analysis.

### 2.2. Clinical and Echocardiographic Variables

Patient characteristics, including clinical parameters, medical history, medications, operative details, and postoperative outcomes, were extracted from electronic medical records. For specific definitions, see the “Definition of variables” in supplementary materials. Echocardiographic variables were extracted from preoperative echocardiographic reports [15].

### 2.3. Coronary Angiography

Preoperative coronary angiograms within 6 months of surgery were acceptable. CAD burden was evaluated according to the percentage of luminal stenosis in coronary arteries > 1.5 mm in diameter. Patients exhibiting 50–70% luminal stenosis in any major epicardial coronary vessels, including their side branches, were classified into the 50–70% group. Conversely, patients with all coronary vessels demonstrating less than 50% stenosis were categorized into the <50% group [16]. As left main (LM) stenosis > 50% and other coronary stenosis > 70% share a similar recommendation class in guidelines, such patients underwent revascularization at our center and were therefore excluded from this study [2].

#### Study Outcomes

The primary outcome was MACE, and the secondary endpoint was all-cause death. MACE was defined as acute coronary syndrome (ACS), stroke, unstable angina requiring hospital admission, or unplanned coronary revascularization (percutaneous or surgical).

### 2.4. Statistical Analysis

Baseline characteristics and perioperative outcomes were compared between study groups using a two-sided Student’s *t*-test, or Mann–Whitney U test for continuous variables and χ^2^ test for categorical variables. Continuous variables are represented as mean (standard deviation) or median (interquartile range), and categorical variables are reported as proportions.

To reduce confounding factors, we used a propensity matching score (PSM) for 1:1 matching. Variables included in the propensity score model were age, gender, eGFR, elevated TG, elevated TC, elevated LDL, smoke, drink, hypertension, diabetes, previous stroke, and EF < 35%.

Follow-up was calculated from the time of surgery to death or the last follow-up. Log-rank test for comparison between groups before and after PSM.

Univariable and multivariable Cox proportional hazards models were used to identify predictors of endpoints in all patients. Parameters considered for selection were determined a priori and included age, male sex, BMI, smoke, drink, hypertension, diabetes, stroke, atrial fibrillation, hyperuricemia or gout, abnormal lung function, previous cardiac surgery, LVEF < 35%, regurgitation, minimal invasive approach, surgery type, percent coronary stenosis (i.e., 50–70% or <50%). Multivariable Cox regression incorporating indicators that reached statistical significance in univariate analysis (*p* < 0.1).

Since death was a competing risk for MACE, we used the cumulative incidence function (CIF) and plotted cumulative incidence curves. The Fine–Gray test was taken between groups. Univariable and multivariable competitive risk regression were conducted. Univariate variables were selected and included in the multivariate analysis strategy, as was the case in the COX regression.

R version 4.4.1 (R Foundation for Statistical Computing, Vienna, Austria) was used for statistical analysis.

## 3. Results

### 3.1. Baseline Characteristics

Table 1 outlines baseline characteristics of patients, stratified by the extent of CAD. In comparison with the <50% group, the 50–70% group had a higher proportion of elderly patients and males, a higher prevalence of hypertension, diabetes, and elevated LDL, a lower eGFR and LVEF, and a larger left ventricular end-diastolic diameter (LVDd) and left ventricular end-systolic diameter (LVDs). After PSM, the baseline between the two groups reached an acceptable balance. SMD was shown in Supplementary Figure S1. Supplementary Table S2 describes the segmental distribution of coronary occlusion in the 50–70% group.

### 3.2. Perioperative Outcomes

All patients underwent aortic valve surgery under cardiopulmonary bypass. Table 2 describes the operative details and postoperative outcomes between groups. Except for the extubation time (*p* = 0.013), no statistical difference was found between the two groups. In the PSM cohort, no statistical difference was found between the two groups.

### 3.3. Mid-Term Survival

**Figure 1:**
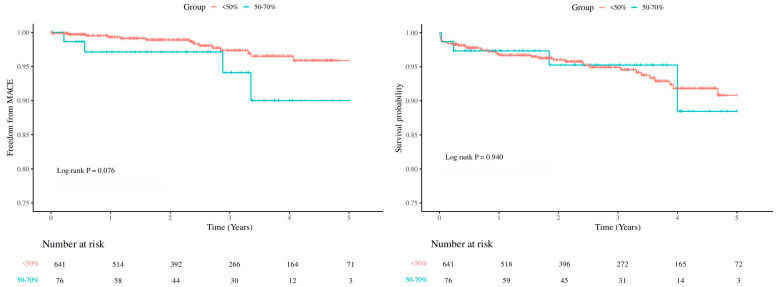
Kaplan–Meier Curve of MACE and survival by coronary artery obstruction degree at 5-year follow-up. Mace, major adverse cardiovascular events.

**Figure 2:**
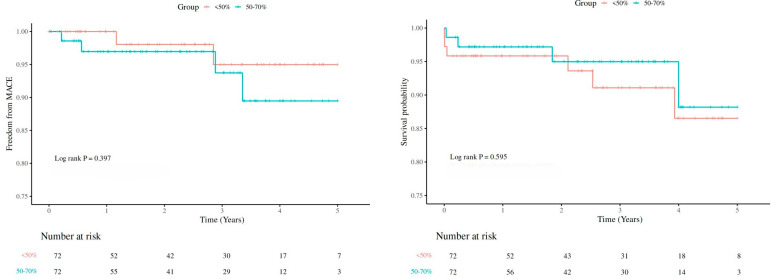
Kaplan–Meier Curve of MACE and survival after PSM by coronary artery obstruction degree at 5-year follow-up. MACE, major adverse cardiovascular events.

Median follow-up time was 2.53 years (IQR: 1.30–3.97 years). Figure 1 shows no difference between the <50% group and 50–70% group in MACE and survival (MACE: *p* = 0.076; all-cause death: *p* = 0.940). Furthermore, no endpoint disparities were identified in the PSM cohort (MACE: *p* = 0.397; all-cause death: *p* = 0.595) (Figure 2). After adjusting for competing risks, the disparity between groups approached no statistical significance (*p* = 0.088) with respect to MACE. Supplementary Table S1 describes the segmental distribution of coronary occlusion in the 50–70% group.

For MACE, it is noteworthy that among the <50% group, three patients experienced recurrent revascularization, unstable angina requiring hospitalization, or postoperative acute coronary syndrome (ACS), while the remaining patient suffered a stroke (Supplementary Table S2). In one case, ACS occurred following surgery, ultimately leading to the patient’s death. In case of recurrent revascularization, following an initial finding of 40% LAD stenosis, this patient presented with chest pain two years later; angiography then showed 80% stenosis, and PCI was performed. For a patient with unstable angina requiring hospitalization, the reason remained unclear.

Table 3 describes univariable and multivariable analyses evaluating MACE and all-cause mortality. Compared with the <50% group, 50–70% coronary obstruction was not associated with all-cause mortality or MACE. Complete results are available in Supplementary Tables S4–S6.

The medication was documented during the follow-up period. It was observed that the proportion of patients taking antithrombotic drugs and statins increased in comparison with the preoperative period, and this increase was more pronounced in the high-load group than in the low-load group (Supplementary Table S3). This was because some patients with no preoperative CAD findings were discharged from the hospital to receive standard coronary care.

## 4. Discussion

The present analysis first demonstrated the procedural safety of aortic surgery for regurgitation in recipients with 50–70% coronary obstruction. We found a very low rate of perioperative complications, all-cause death, and MACE over the total follow-up period.

Our findings suggest that the 5-year incidence of MACE is low in patients with AR. However, our challenge to the mandatory 50% revascularization principle requires further validation [17].

Our results are different from those published by Thalji et al. [3]. This discrepancy may be explained by two key distinctions. First, our cohort consisted exclusively of patients with AR and did not include those with AS, which often shares atherosclerotic pathways with CAD [18]. Second, our study incorporated MACE as an endpoint and systematically recorded postoperative pharmacotherapy, including statin use, which was more common in the high-load group and may have a positive effect on outcomes [19,20]. Our conclusions are similar to those of Forno, namely that omitting coronary artery bypass grafting is safe in patients with moderate coronary artery obstruction whose primary indication is valve surgery [5]. At the same time, the rate of coronary events in post-operative patients was lower than in patients with a similar degree of isolated coronary artery disease [21,22,23]. This may suggest that patients with AR have increased coronary perfusion after correction of valvular regurgitation.

In a study based on quantitative blood flow ratio (QFR)-guided CABG, about half of the coronary lesions between 70% and 90% (visually estimated) and over 60% coronary lesions between 50% and 70% (visually estimated) were QFR-negative. Functional assessment could be a more precise way of measuring, associated with less grafting and better clinical outcome [24,25,26]. However, coronary blood flow is altered in the setting of concomitant valvular heart disease (VHD) [2]. Growing evidence suggests that functional indices remain reliable in aortic stenosis despite pressure overload [27,28]. Whether these findings extend to aortic regurgitation, however, requires further investigation. Cardiovascular Magnetic Resonance (CMR) can help assess ischemia and myocardial viability, which may better identify high-risk CAD phenotypes and guide revascularization decisions [29].

This study suggests that concomitant CABG is performed under more demanding conditions in patients with AR. Concomitant CABG is a viable option for patients with a higher disease burden and more severe angina symptoms that appear to be more attributable to coronary artery disease than valve disease.

Several limitations of the present analysis warrant further consideration. This study was a single-center retrospective study. Given the large catchment area of our center, there could have been instances of inconsistent follow-up, as patients typically return to their local institutions for more routine care post-surgery. This could have resulted in an underestimation of events captured post-surgery. Our method of assessing risk categories was based on the vessel affected and the degree of occlusion, representative of clinical workflow. The latest guideline seems to still place hope on using coronary functional assessments, such as instantaneous wave-free ratio (iFR) or fractional flow reserve (FFR) [17]. Unlike patients with isolated CAD, myocardial oxygen demand increases due to ventricular remodeling caused by late-stage valve disease, and it is more accurate to include both oxygen supply and demand in the assessment, which can also be challenging. Given that clinical coronary angiography is currently the most widely used and analyzed with negative results, it can be argued that reliance on it can provide convenient and economical clinical guidance.

## 5. Conclusions

In patients with aortic insufficiency, 50–70% chronic coronary obstruction does not increase perioperative complications, MACE, and all-cause mortality.

## Figures and Tables

**Table 1:** Baseline characteristics according to the extent of CAD before and after PSM.

Variables	Before PSM					After PSM				
	Total (*n* = 717)	50–70% (*n* = 76)	<50% (*n* = 641)	*p*	SMD	Total (*n* = 144)	50–70% (*n* = 72)	<50% (*n* = 72)	*p*	SMD
**Clinical parameters**										
Age, years	64.00 (56.00, 69.00)	68.00 (64.00, 73.00)	63.00 (56.00, 69.00)	**<0.001**	0.651	68.00 (62.00, 73.00)	68.00 (61.75, 73.00)	67.50 (63.75, 73.00)	0.994	0.009
Male	458 (63.88)	58 (76.32)	400 (62.40)	**0.017**	0.327	114 (79.17)	60 (83.33)	54 (75.00)	0.218	0.192
BMI, kg/m^2^	23.44 (21.31, 25.71)	23.16 (20.85, 25.79)	23.46 (21.33, 25.64)	0.558	0.111	22.97 (20.87, 25.82)	22.90 (20.91, 26.25)	23.16 (20.85, 25.79)	0.920	0.102
**Past medical history**										
Smoke	134 (18.69)	20 (26.32)	114 (17.78)	0.071	0.194	38 (26.39)	19 (26.39)	19 (26.39)	1.00	0.000
Drink	107 (14.92)	14 (18.42)	93 (14.51)	0.365	0.101	27 (18.75)	14 (19.44)	13 (18.06)	0.831	0.036
Hypertension	370 (51.60)	51 (67.11)	319 (49.77)	**0.004**	0.369	98 (68.06)	51 (70.83)	47 (65.28)	0.475	0.117
Diabetes	53 (7.39)	14 (18.42)	39 (6.08)	**<0.001**	0.318	19 (13.19)	8 (11.11)	11 (15.28)	0.460	0.116
Insulin,	13 (1.81)	2 (2.63)	11 (1.72)	0.912	0.057	3 (2.08)	1 (1.39)	2 (2.78)	1.000	0.119
Stroke	64 (8.93)	8 (10.53)	56 (8.74)	0.605	0.058	17 (11.81)	10 (13.89)	7 (9.72)	0.438	0.141
Atrial fibrillation	169 (23.57)	17 (22.37)	152 (23.71)	0.794	0.032	29 (20.14)	13 (18.06)	16 (22.22)	0.533	0.108
Hyperuricemia or gout	17 (2.37)	0 (0.00)	17 (2.65)	0.299	0.175	2 (1.39)	2 (2.78)	0 (0.00)	0.476	0.169
Renal insufficiency	25 (3.49)	3 (3.95)	22 (3.43)	1.000	0.026	5 (3.47)	2 (2.78)	3 (4.17)	1.000	0.085
Abnormal lung function	28 (3.91)	2 (2.63)	26 (4.06)	0.769	0.089	4 (2.78)	2 (2.78)	2 (2.78)	1.000	0.000
Dialysis	5 (0.70)	0 (0.00)	5 (0.78)	1.000	0.094	0 (0.00)	0 (0.00)	0 (0.00)	1.000	0.000
**Medications**										
Antiplatelet agent	22 (3.07)	3 (3.95)	19 (2.96)	0.906	0.050	5 (3.47)	2 (2.78)	3 (4.17)	1.000	0.085
Anticoagulants	38 (5.30)	0 (0.00)	38 (5.93)	0.056	0.265	5 (3.47)	5 (6.94)	0 (0.00)	0.069	0.273
Statins	11 (1.53)	1 (1.32)	10 (1.56)	1.000	0.021	2 (1.39)	1 (1.39)	1 (1.39)	1.000	0.000
**Labs**										
eGFR, mL/min/1.73 m^2^				**0.013**					0.960	
≥90	523 (72.94)	44 (57.89)	479 (74.73)		0.341	86 (59.72)	44 (61.11)	42 (58.33)		0.056
60–89	160 (22.32)	27 (35.53)	133 (20.75)		0.309	49 (34.03)	24 (33.33)	25 (34.72)		0.029
30–59	26 (3.63)	4 (5.26)	22 (3.43)		0.082	7 (4.86)	3 (4.17)	4 (5.56)		0.061
15–29	3 (0.42)	1 (1.32)	2 (0.31)		0.088	2 (1.39)	1 (1.39)	1 (1.39)		0.000
≤15	5 (0.70)	0 (0.00)	5 (0.78)		0.094	0 (0.00)	0 (0.00)	0 (0.00)		0.000
Elevated TG	38 (5.30)	2 (2.63)	36 (5.62)	0.408	0.186	2 (1.39)	0 (0.00)	2 (2.78)	0.476	0.169
Elevated TC	10 (1.39)	1 (1.32)	9 (1.40)	1.000	0.008	2 (1.39)	1 (1.39)	1 (1.39)	1.000	0.000
Elevated LDL	161 (22.45)	62 (81.58)	99 (15.44)	**<0.001**	0.135	13 (9.03)	6 (8.33)	7 (9.72)	0.771	0.047
**Echocardiography**										
LVEF, %	54.00 (46.00, 58.00)	52.00 (42.92, 57.00)	54.00 (46.60, 58.00)	**0.028**	0.271	52.75 (44.08, 58.00)	54.00 (43.58, 58.00)	52.15 (44.45, 57.25)	0.619	0.048
LVEF < 35%	45 (6.28)	10 (13.16)	35 (5.46)	**0.018**	0.228	15 (10.42)	8 (11.11)	7 (9.72)	0.785	0.047
LVDs, cm	4.35 (3.80, 5.10)	4.80 (4.15, 5.54)	4.30 (3.77, 5.05)	**0.002**	0.432	6.30 (5.75, 7.10)	6.10 (5.57, 7.05)	6.42 (5.86, 7.15)	0.210	0.230
LVDd, cm	6.10 (5.50, 6.80)	6.43 (5.90, 7.15)	6.08 (5.45, 6.78)	**0.004**	0.402	4.46 (3.94, 5.43)	4.35 (3.83, 5.40)	4.80 (4.10, 5.50)	0.176	0.222
Regurgitation				0.653					0.834	
Moderate	237 (33.05)	22 (28.95)	215 (33.54)		0.101	45 (31.25)	24 (33.33)	21 (29.17)		0.088
Moderately severe	209 (29.15)	22 (28.95)	187 (29.17)		0.005	44 (30.56)	22 (30.56)	22 (30.56)		0.000
severe	271 (37.80)	32 (42.11)	239 (37.29)		0.098	55 (38.19)	26 (36.11)	29 (40.28)		0.087

Values are presented as mean ± standard deviation, n (%), or median (interquartile range). Bold is meant to highlight significant values below a threshold of *p* = 0.05. PSM, propensity score matching; CAD, coronary artery disease; BMI, body mass index; eGFR, estimated glomerular filtration rate; TG, triglyceride; TC, total cholesterol; LDL, low-density lipoprotein; LVEF, left ventricular ejection fraction; LVDs, left ventricular end-diastolic diameter; LVDd, left ventricular end-systolic diameter. Bold font indicates that the *p*-value is less than 0.05.

**Table 2:** Operative details and postoperative outcomes according to the extent of CAD before and after PSM.

Variables	Before PSM				After PSM			
	Total (*n* = 717)	50–70% (*n* = 76)	<50% (*n* = 641)	*p*	Total (*n* = 144)	50–70% (*n* = 72)	<50% (*n* = 72)	*p*
**Operative details**								
Minimal Invasive Approach	183 (25.52)	16 (21.05)	167 (26.05)	0.344	34 (23.61)	18 (25.00)	16 (22.22)	0.695
Surgery Type				0.421				0.594
Isolated AVR or AVP	164 (22.87)	13 (17.11)	151 (23.56)		31 (21.53)	18 (25.00)	13 (18.06)	
Multi-valve	301 (41.98)	33 (43.42)	268 (41.81)		60 (41.67)	29 (40.28)	31 (43.06)	
Combined aorta	252 (35.15)	30 (39.47)	222 (34.63)		53 (36.81)	25 (34.72)	28 (38.89)	
CPB, min	140.00 (110.00, 177.00)	133.50 (109.75, 173.00)	141.00 (110.00, 178.00)	0.406	138.00 (110.00, 173.00)	139.00 (116.50, 172.50)	135.00 (109.75, 173.00)	0.460
ACC, min	105.00 (82.00, 138.00)	99.00 (82.00, 130.25)	106.00 (82.00, 139.00)	0.258	102.00 (82.00, 130.25)	103.50 (82.00, 129.00)	101.00 (83.50, 130.25)	0.538
24h drainage, mL	350.00 (220.00, 500.00)	300.00 (230.00, 475.00)	350.00 (220.00, 510.00)	0.282	320.00 (230.00, 500.00)	380.00 (225.00, 585.00)	300.00 (230.00, 492.50)	0.357
Extubation, hours	8.00 (5.50, 15.00)	12.00 (7.00, 17.00)	8.00 (5.30, 14.50)	**0.013**	10.50 (6.00, 16.12)	10.00 (5.50, 15.62)	12.00 (6.75, 17.00)	0.434
ICU, hours	50.00 (44.00, 71.00)	65.00 (45.00, 70.00)	49.00 (44.00, 72.00)	0.385	64.00 (44.00, 72.00)	48.50 (44.00, 87.50)	65.00 (45.00, 70.00)	0.754
Hospital length after surgery, days	11.00 (9.00, 14.00)	11.00 (9.00, 13.25)	11.00 (9.00, 14.00)	0.384	11.00 (9.00, 14.00)	11.00 (9.00, 14.00)	11.00 (9.00, 13.00)	0.786
LOS, day	18.00 (15.00, 22.00)	19.00 (16.00, 22.00)	18.00 (15.00, 22.00)	0.424	18.50 (16.00, 22.00)	19.00 (16.00, 22.25)	18.00 (16.00, 22.00)	0.377
**Postoperative outcomes**								
IABP	9 (1.26)	1 (1.32)	8 (1.25)	1.000	2 (1.39)	1 (1.39)	1 (1.39)	1.000
CRRT	11 (1.53)	0 (0.00)	11 (1.72)	0.511	1 (0.69)	1 (1.39)	0 (0.00)	1.000
ECMO	4 (0.56)	1 (1.32)	3 (0.47)	0.362	1 (0.69)	0 (0.00)	1 (1.39)	1.000
Pleural effusion/Pericardial effusion	128 (17.85)	11 (14.47)	117 (18.25)	0.416	29 (20.14)	18 (25.00)	11 (15.28)	0.146
Cardiac arrest defibrillation	3 (0.42)	0 (0.00)	3 (0.47)	1.000	0 (0.00)	0 (0.00)	0 (0.00)	1.000
Secondary thoracotomy	14 (1.95)	1 (1.32)	13 (2.03)	1.000	1 (0.69)	0 (0.00)	1 (1.39)	1.000
New-onset atrial fibrillation	18 (2.51)	0 (0.00)	18 (2.81)	0.275	2 (1.39)	2 (2.78)	0 (0.00)	0.476
Electrical cardioversion	81 (11.30)	6 (7.89)	75 (11.70)	0.322	14 (9.72)	10 (13.89)	4 (5.56)	0.091
Secondary-intubation/Tracheostomy	6 (0.84)	1 (1.32)	5 (0.78)	0.491	1 (0.69)	0 (0.00)	1 (1.39)	1.000
Incision infection/Poor healing/Mediastinal infection	10 (1.39)	0 (0.00)	10 (1.56)	0.562	1 (0.69)	1 (1.39)	0 (0.00)	1.000
Perioperative cerebral infarction	3 (0.42)	0 (0.00)	3 (0.47)	1.000	0 (0.00)	0 (0.00)	0 (0.00)	1.000
Perioperative myocardial infarction	0 (0.00)	0 (0.00)	0 (0.00)	1.000	0 (0.00)	0 (0.00)	0 (0.00)	1.000
Peri-procedural death	11 (1.53)	1 (1.32)	10 (1.56)	1.000	4 (2.78)	3 (4.17)	1 (1.39)	0.612

PSM, propensity score matching; AVR, aortic valve replacement; AVP, Aortic valve repair; CPB, cardiopulmonary bypass; ACC, aortic cross clamp; ICU, intensive care unit; LOS, length of stay; IABP, intra-aortic balloon pump; CRRT, continuous renal replacement therapy; ECMO, extracorporeal membrane oxygenation. Bold font indicates that the *p*-value is less than 0.05.

**Table 3:** Univariable and multivariable Cox proportional-hazard regression and competing risk regression of all-cause mortality and major adverse cardiovascular events.

Group	Unadjusted		Multivariable Adjusted	
	HR (95% CI)	*p*-Value	HR (95% CI)	*p*-Value
**COX regression**				
**MACE**				
<50%	Ref	N/A	Ref	N/A
50–70%	2.64 (0.87~8.04)	0.088	1.65 (0.51~5.37)	0.402
**All-cause mortality**				
<50%	Ref	N/A	N/A	N/A
50–70%	1.04 (0.37~2.93)	0.940	N/A	N/A
**Competing risk regression**				
**MACE**				
<50%	Ref	N/A	Ref	N/A
50–70%	2.66 (0.89~7.99)	0.081	1.58 (0.44~5.70)	0.49

CI, confidence interval; HR, hazard ratio; N/A, not available; MACE, major adverse cardiovascular events.

## Data Availability

The raw data supporting the conclusions of this article will be made available by the authors on request.

## Supplementary Materials

The following supporting information can be downloaded at: https://www.mdpi.com/article/10.3390/jcdd13030115/s1. Supplementary Table S1. Segmental distribution of coronary artery 50–70% obstruction; Supplementary Table S2. Specific MACE event; Supplementary Table S3. Medication adherence during follow-up; Supplementary Table S4. Multivariable COX regression of factors associated with MACE in the whole cohort; Supplementary Table S5. Multivariable COX regression of factors associated with All-cause mortality in the whole cohort; Supplementary Table S6. Multivariable competitive risk regression of factors associated with MACE in the whole cohort; Supplementary Figure S1. Standardized mean difference (SMD) before and after matching showed that there was a decrease in the SMD values.

## Abbreviations

The following abbreviations are used in this manuscript:

CABG	Coronary artery bypass grafting
AVR	Aortic valve replacement
AS	Aortic stenosis
AR	Aortic regurgitation
CAD	Coronary artery disease
MACE	Major adverse cardiovascular events
PCI	Percutaneous coronary intervention
LVEF	Left ventricular ejection fraction
LAD	Left anterior descending artery
LCX	Left circumflex artery
RCA	Right coronary artery
