# Supplementary material for: Prognostic Impact of Untreated Chronic Coronary Artery Obstruction After Surgery for Aortic Regurgitation
Source: J Cardiovasc Dev Dis. 2026 Mar 3;13(3):115. doi: 10.3390/jcdd13030115 (PMC13027027; doi:10.3390/jcdd13030115)

Definition of variables

Individuals who smoked ⩾ 100 cigarettes in their lifetime were defined as "smokers", which included ever smokers and current smokers. Subjects with alcohol consumption at least once a week for ⩾ 1 year were defined as "alcohol drinkers". One drink was defined as 375 ml of beer (13.6 g of ethanol), 118 ml of wine (11.7 g of ethanol), or 30 ml of western or Chinese hard liquor (10.9 g of ethanol). Elevated LDL is defined as LDL > 3.4 mmol/L. Elevated TG is defined as TG > 2.3 mmol/L. Elevated TC is defined as TC > 6.2mmol/L[1]. Abnormal lung function is defined as severe pulmonary ventilation impairment.

Supplementary Table S1. Segmental distribution of coronary artery 50-70% obstruction.

Coronary segmentation	50-70% obstruction
pLAD	18 (16.07)
mLAD	28 (25.00)
dLAD	7 (6.25)
D1	7 (6.25)
D2	1 (0.89)
pLCX	8 (7.14)
mLCX	8 (7.14)
dLCX	7 (6.25)
OM	1 (0.89)
pRCA	9(8.04)
mRCA	10 (8.93)
dRCA	4 (3.57)
PDA	1 (0.89)
PLB	3 (2.68)

Values are presented as mean ± standard deviation, n (%), or median (interquartile range).

pLAD, proximal left anterior descending artery; mLAD, middle left anterior descending artery; dLAD, distal left anterior descending artery; pLCX, proximal left circumflex artery; mLCX, middle left circumflex artery; dLCX, distal left circumflex artery; pRCA, proximal right coronary artery; mRCA, middle right coronary artery; dRCA, distal right coronary artery; D1, first diagonal branches; D2, second diagonal branches; OM, obtuse marginal; PDA, posterior descending artery; PLB, posterior lateral branch.

Supplementary Table S2. Specific MACE event.

Variables	Before PSM			After PSM		
	**Total** (n = 717)	**50-70%** (n = 76)	**<50%** (n = 641)	**Total** (n = 144)	**50-70%** (n = 72)	**<50%** (n = 72)
**MACE**	22	4	18	6	4	2
Acute coronary syndrome	1	0	1	1	0	1
Stroke	19	4	15	5	4	1
Unstable angina requiring hospital admission	1	0	1	0	0	0
Unplanned coronary revascularization (percutaneous or surgical)	1	0	1	0	0	0

Supplementary Table S3. Medication adherence during follow-up

Variables	Before PSM				After PSM			
	Total (n = 717)	50-70% (n = 76)	<50% (n = 641)	P	Total (n = 144)	50-70% (n = 72)	<50% (n = 72)	P
**ACEI /Calcium channel blockers**	138 (19.25)	14 (18.42)	124 (19.34)	0.847	34 (23.61)	13 (18.06)	21 (29.17)	0.116
**Beta-blockers**	98 (13.67)	12 (15.79)	86 (13.42)	0.569	22 (15.28)	12 (16.67)	10 (13.89)	0.643
**Diuretics**	34 (4.74)	3 (3.95)	31 (4.84)	0.953	9 (6.25)	3 (4.17)	6 (8.33)	0.491
**Antihyperglycemic agent**	30 (4.18)	6 (7.89)	24 (3.74)	0.160	12 (8.33)	6 (8.33)	6 (8.33)	1.000
**Antithrombins**	169 (23.57)	21 (27.63)	148 (23.09)	0.378	34 (23.61)	21 (29.17)	13 (18.06)	0.116
**Statins**	64 (8.93)	11 (14.47)	53 (8.27)	0.073	17 (11.81)	11 (15.28)	6 (8.33)	0.197

Values are presented as mean ± standard deviation, n (%), or median (interquartile range).

Bold is meant to highlight significant values below a threshold of p = .05

ACEI, angiotensin converting enzyme inhibitors.

Supplementary Table S4. Multivariable COX regression of factors associated with MACE in whole cohort

Variable	HR (95% CI)	P-value
**Age**	1.08 (1.01 ~ 1.15)	0.017

CI, confidence interval; HR, hazard ratio;

Supplementary Table S5. Multivariable COX regression of factors associated with All-cause mortality in whole cohort

Variable	HR (95% CI)	P-value
**Abnormal lung function**	2.99 (1.14 ~ 7.83)	0.026
**LVEF<35%**	3.82 (1.70 ~ 8.58)	0.001
**Age**	1.07 (1.03 ~ 1.12)	<.001

CI, confidence interval; HR, hazard ratio;

Supplementary Table S6. Multivariable competitive risk regression of factors associated with MACE in whole cohort

Variable	HR (95% CI)	P-value
**Age**	1.07 (1.00 ~ 1.14)	0.036

CI, confidence interval; HR, hazard ratio;

Supplementary Figure S1. Standardized mean difference (SMD) before and after matching, showed that there was a decrease in the SMD values.

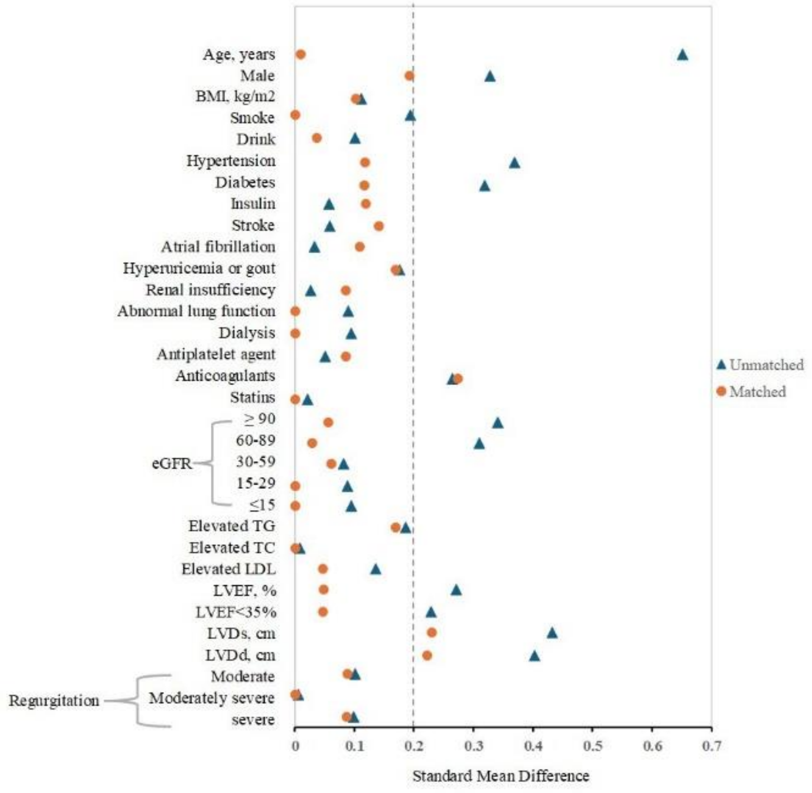

Supplementary Figure S2. Study flow chart.

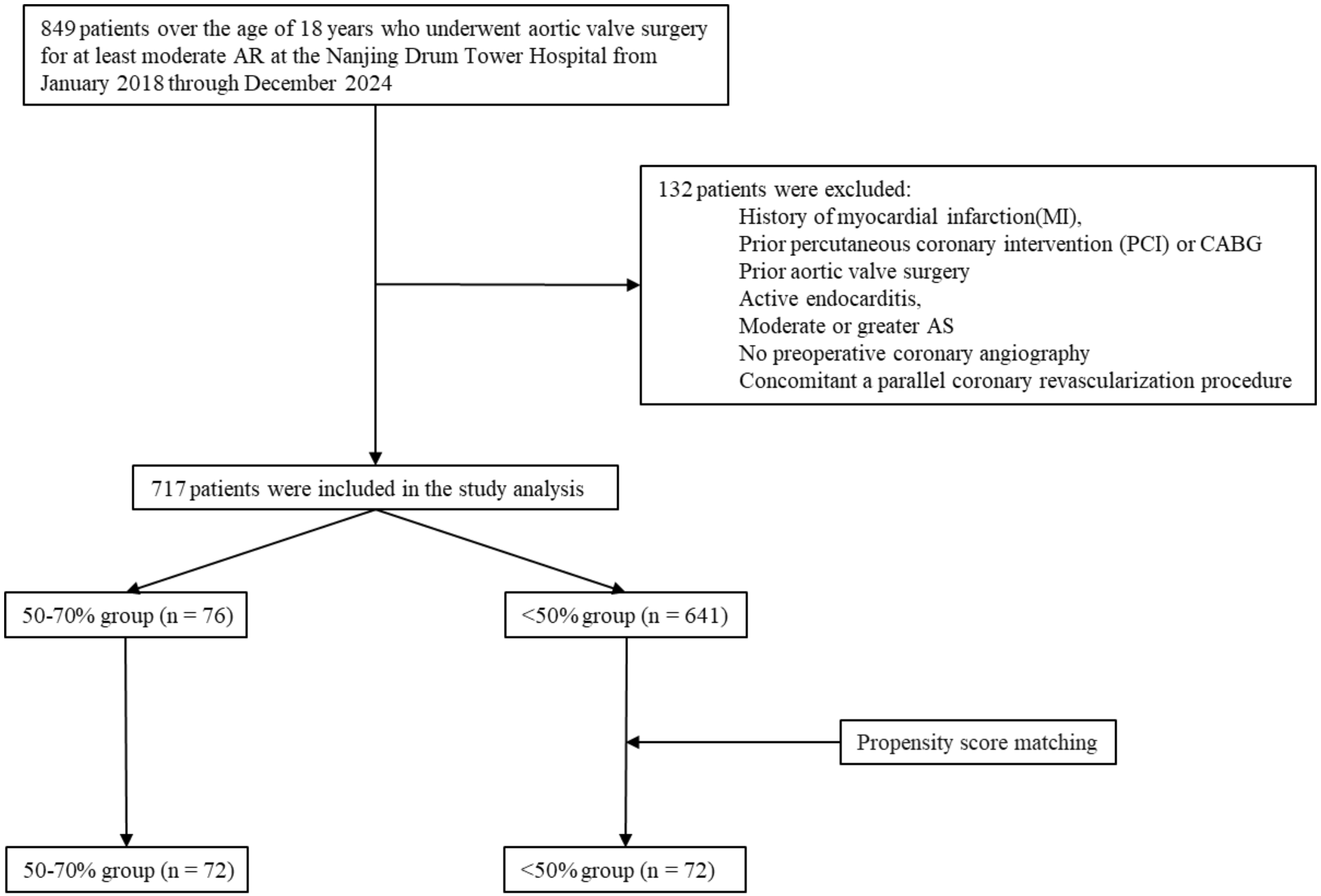

Supplement: Supplementary file 1 [file jcdd-13-00115-s001.zip › jcdd-4131786-supplementary.pdf]
